# Experimental Analysis of MOC Composite with a Waste-Expanded Polypropylene-Based Aggregate

**DOI:** 10.3390/ma11060931

**Published:** 2018-05-31

**Authors:** Martina Záleská, Milena Pavlíková, Ondřej Jankovský, Michal Lojka, Adam Pivák, Zbyšek Pavlík

**Affiliations:** 1 Department of Materials Engineering and Chemistry, Faculty of Civil Engineering, Czech Technical University in Prague, Thákurova 7, 166 29 Prague 6, Czech Republic; martina.zaleska@fsv.cvut.cz (M.Z.); milena.pavlikova@fsv.cvut.cz (M.P.); adam.pivak@fsv.cvut.cz (A.P.); 2 Department of Inorganic Chemistry, Faculty of Chemical Technology, University of Chemistry and Technology, Technická 5, 166 28 Prague 6, Czech Republic; ondrej.jankovsky@vscht.cz (O.J.); michal.lojka@vscht.cz (M.L.)

**Keywords:** magnesium oxychloride cement, waste expanded polypropylene, ultra-lightweight aggregate, thermal insulation, mechanical parameters, water resistance

## Abstract

Polypropylene (PP) is one of the most widely produced types of plastic worldwide, but its recycling is limited. This work presents a study of the utilization of expanded polypropylene (EPP) waste in a magnesium oxychloride cement (MOC) composite usable in the building industry. MOC is formed by mixing magnesium oxide powder and a concentrated solution of magnesium chloride and is characterized by excellent bonding ability to large quantities of different types of aggregates. A developed air-cured MOC composite, where an EPP-based aggregate was used for the full replacement of natural aggregate, was investigated in terms of its basic physical, mechanical, thermal and water resistance properties. The results demonstrate that incorporation of EPP waste greatly improved the thermal insulation properties, while the mechanical resistance was reduced to an acceptable level. The developed MOC composite containing EPP waste can be considered as an alternative thermal insulation material applicable for the construction of floor or envelope construction systems.

## 1. Introduction

**Figure 1:**
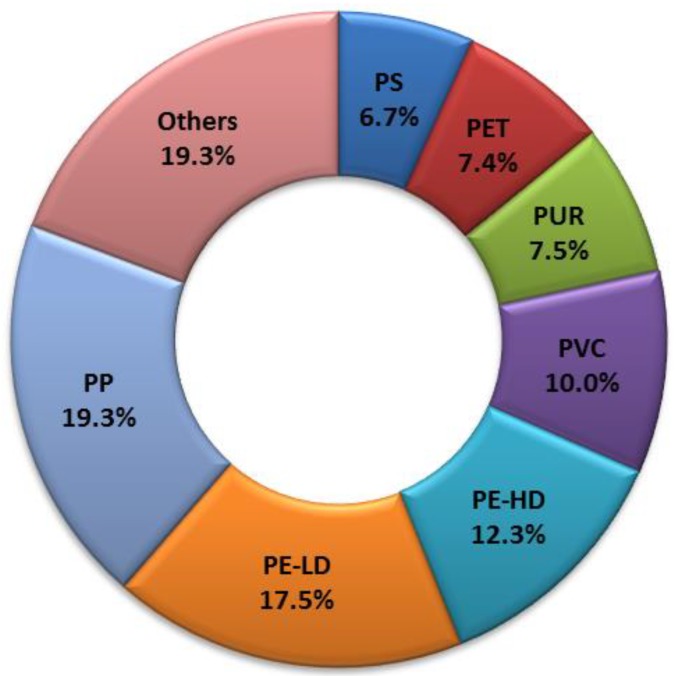
Plastic demand in Europe in 2016 per polymer type [2]; PP—polypropylene, PS—polystyrene, PET—polyethylene terephthalate, PUR—polyurethane, PVC—polyvinylchloride, PE-HD—high density polyethylene, PE-LD—low density polyethylene.

As a result of the use of plastics in a wide range of products, a huge amount of plastic waste is being generated every year. The consumption of plastics is readily increasing due to their various advantages such as versatility, low cost and high chemical stability [1]. Plastic production data from 2016 show the global production of 335 Mt and the European production of 60 Mt. Considering the types of polymers that make up the bulk of the collected plastic waste, a fair idea can be obtained by looking at the plastic demand for new products. In terms of plastic demand by polymer type (Figure 1), Europe was the leader in 2016 with respect to polypropylene (PP), with 19.3% [2,3].

The preferred option in waste management is recycling. In recycling, new raw materials are obtained via a mechanical or chemical pathway. If polymer waste cannot be recycled, energy recovery is the preferred option. Landfills, the least preferred option, should be avoided at all cost [3]. In Europe, in ten years (2006–2016), plastic waste recycling has increased by almost 80%, and for the first time, in 2016, more plastic waste was recycled (31.1%) than landfilled (27.3%). However, due to the ban on landfilling of plastic waste in some European countries, recycling is unevenly distributed among the states of Europe [2]. Furthermore, every type of plastic waste is recycled to a different extent. As we can see, PP represents the largest part of plastic production, but its recycling ratio is very low. For example, in the United States in 2012, when PP in plastic production reached second place with 22.6%, its recycling ratio was only 0.6%, which makes it one of the least recycled postconsumer plastics [4].

In 2010, buildings accounted for 32% of global final energy use. Increase in energy consumption in buildings in the last twenty years has been modest in US and EU countries, due to the improvement of energy efficiency and the promotion of renewable energy sources. The ambitious EU 2030 Climate & Energy package fixed the reduction of greenhouse gas emissions at 40% from 1990 levels, the share for renewable energy at 27% and improvement in energy efficiency at 27% [5]. Special attention is devoted to buildings due to the large amount both of energy consumption and estimated energy savings of this sector in EU member states. Technology innovations offer new solutions for buildings and smart energy concepts that enable more dynamic and interactive buildings, where technologies are used in conjunction with optimum design techniques [6,7,8]. On the other hand, as the economy, urbanization, and population of developing countries continue to grow, a significant increase in building energy consumption in these countries is expected. Given the current global demand trends, building energy saving is highly critical [9,10]. In order to design buildings with preferable thermal attributes and optimum energy performance, an important element is the envelope design, and the use of materials with effective thermal insulation properties should be taken into consideration [10,11].

Among the various types of recycling management approaches, the reuse of waste and recycled plastic material in the construction industry is considered an ideal method for disposing plastic waste [8]. The great advantages of this method include that the recycled plastics substitute the use of virgin construction materials (with related environmental and energy impacts) and moreover, in terms of energy saving, the composites with plastic aggregates exhibit improved thermal insulation performance. The use of recycled plastic materials in conventional cement mortar and concrete has been researched extensively [8,9,10,11,12,13,14]. On the other hand, no study has reported the use of waste plastic-based aggregates in MOC composites to date. 

As an air-dried magnesia-based cementing material, magnesium oxychloride cement (MOC) was developed by a French scientist, Sorel, shortly after the invention of Portland cement in 1867 [15,16,17]. It is obtained by mixing magnesium oxide (MgO) powder with a concentrated solution of magnesium chloride (MgCl_2_) and is one of the main representative chemically bonded cements [18,19,20]. Through a solution reaction, two chemical composition phases, namely, phase 3 (3Mg(OH)_2_·MgCl_2_·8H_2_O) and phase 5 (5Mg(OH)_2_·MgCl_2_·8H_2_O), can be formed at ambient temperature and provide the bulk of the mechanical integrity [21]. The production of lightly burnt MgO used in MOC requires a much lower calcination temperature compared to that for Portland cement, thus reducing the vast amount of energy consumption [22]. 

MOC has been reported with many superior properties over Portland cement, such as high mechanical strength [22], good fire resistance [23,24,25], low thermal conductivity [21,26], resistance to abrasion [20,27] and especially remarkable high bonding ability to different types of fillers [19,21,22]. By virtue of these advantages, MOC has been well utilized for making floors, fire protection panels, thermal insulation composites, decoration panels, grinding wheels, etc. [21,22].

Taking into account all the above-mentioned facts and knowledge gaps, the present study is aimed at evaluation of the use of waste-expanded polypropylene aggregate as a full replacement of natural aggregate for the development of MOC composite. In terms of both waste management and the improvement of material thermal resistance, the main objective was to produce an MOC composite incorporating as much waste plastic aggregate as possible, to obtain material with enhanced thermal properties that still meets the requirements for workability and strength. Based on literature analysis, such modification of MOC composite composition has not been reported, and the present study can therefore be considered as an initial attempt in this manner. Investigation of an MOC composite with incorporated waste-expanded polypropylene was performed in order to determine its mechanical, physical and thermal properties and water resistance behavior. This new developed material with enhanced thermal insulation performance can find application in the floor and wall structural systems of buildings, can prevent energy loss and will enable the reuse of a large amount of the studied type of plastic waste.

## 2. Materials and Methods 

### 2.1. Material and Sample Preparation

Expanded polypropylene (EPP) in the form of shredded waste from aircraft model production was used in this research. Expanded polypropylene is generally characterized by low weight, high strength-to-weight ratio, thermal and sound insulation properties (even in a moist environment), good chemical resistance, and burning with the absence of non-toxic gas release compared to polystyrene foam [28]. Waste plastic-based aggregate was employed for full replacement of natural silica aggregate and expressed in vol. %, i.e., EPP was applied in an amount equivalent to 150% by volume of silica sand. The amount of EPP-based aggregate in the MOC composite mix was selected on the basis of preliminary tests of fresh composite mixes, where workability of MOC composites with EPP in an amount of 100%, 150% and 200% by volume of silica sand was applied. As given above, the purpose of our design was to develop a lightweight material with good thermal insulation properties and sufficient mechanical strength for nonstructural application in the construction industry. Therefore, it was decided to fully replace silica sand in the mix composition and apply as much of the EPP as possible. Based on flow table test results, the mix with the highest amount of EPP was found to be unworkable with negligible flow. On the other hand, the mix with EPP in an amount of 100% by volume of silica sand had the best workability with a flow of 200 mm. The composite mix with 150% of EPP by volume of silica sand that was studied in detail in the paper showed good rheological performances and was easy to treat and work with. In general, the composition of the tested MOC composite can be considered as limiting from the point of view of the amount of incorporated EPP in a mix.

Caustic magnesite powder was produced by Styromagnesit Steirische Magnesitindustrie Ltd., Oberdorf, Austria. The chemical composition and basic physical properties of the MgO powder are summarized in Table 1 and Table 2. The caustic magnesite powder was milled to an average particle size of about 45 µm.

**Figure 2:**
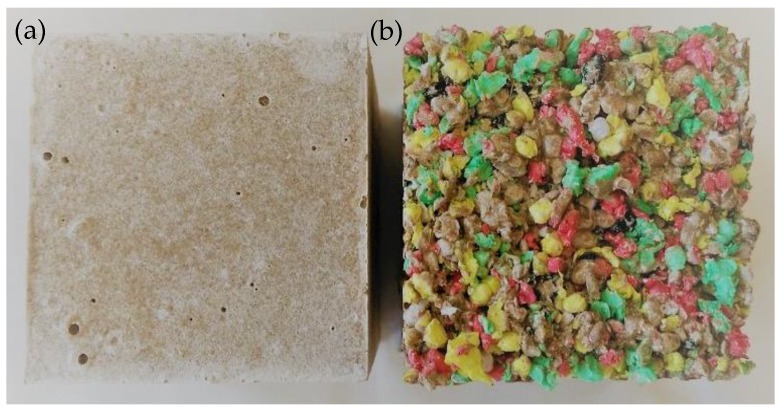
Tested MOC-R (**a**) and MOC-EPP (**b**) samples.

MgCl_2_·6H_2_O of p.a. purity from Lach-Ner Ltd., Neratovice, Czech Republic, was dissolved in tap water to obtain a magnesium chloride solution. The concentration of the prepared solution was expressed in terms of specific gravity on a Baume scale as 26 °Bé. The silica sand used for the preparation of reference samples was supplied by Filtrační písky Ltd., affiliate Chlum u Doks, Czech Republic. Before preparation of the MOC composite mixes, silica sand with a particle size fraction of 0–2 mm, which complies with EN 196-1 [29], was mixed from three sand normalized fractions PG1 (0/0.5 mm), PG2 (0.5/1 mm) and PG3 (1/2 mm). The weight ratio of the particular sand fractions was 1:1:1. The specific gravity of the silica sand was 2652 kg/m^3^. The investigated MOC-based composites were prepared by mixing caustic magnesite powder, MgCl_2_ solution and silica sand (referred to as MOC-R) or EPP aggregate (referred to as MOC-EPP). Mixed proportions of fabricated MOC materials are presented in Table 3. The molar ratio of MgO/MgCl_2_ was 6.9, and the molar ratio of H_2_O/MgCl_2_ was 9.4. The setting time of the MOC-R fresh mixture was determined with an automatic Vicat apparatus (B26600, Form Test Seidner + Co. GmbH, Riedlingen, Gramany); the observed initial setting time was 8.7 h, and the final setting time was 12.6 h. The fresh mixtures were casted in cubic molds with a dimension of 70 × 70 × 70 mm and prisms molds with the size of 40 × 40 × 160 mm. Specimens were demolded after 24 h and air-cured under laboratory conditions (22 ± 2 °C; 45 ± 5% relative humidity) for 27 days. The tested cubic specimens are shown in Figure 2.

### 2.2. Testing Methods

#### 2.2.1. Aggregate Characterization

**Figure 3:**
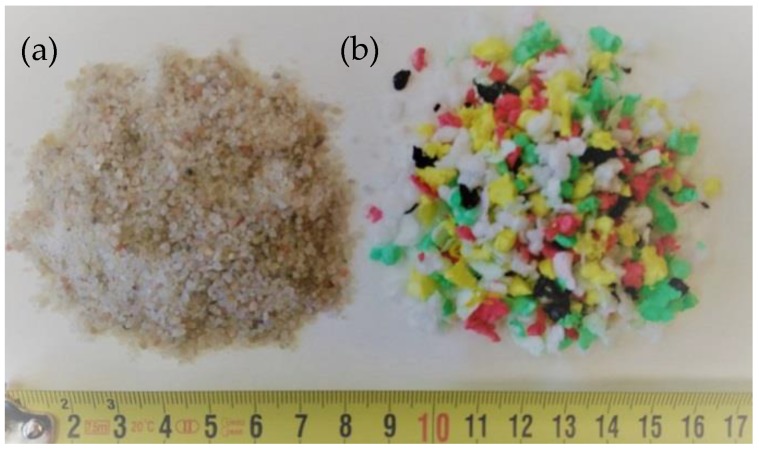
Natural (**a**) and waste-expanded polypropylene-based aggregate (**b**).

For both natural and waste plastic aggregates (Figure 3), their particle size and the thermal transport and storage properties were determined, which were measured with respect to powder density and time of compaction. 

The grain-size curves of aggregates were obtained using a standard sieve method with sieves of the following mesh dimensions: 0.063; 0.125; 0.25; 0.5; 1.0; 2.0; 4.0; and 8.0 mm. In order to evaluate the thermal performance of the studied aggregates, their powder density and thermal properties, which included thermal conductivity *λ* (W/mK) and volumetric heat capacity *C_v_* (J/m^3^K), were measured based on compaction time. During the test, a particular aggregate was inserted into the measuring cylinder and compacted by means of a vibration exciter (VSB 15, BRIO Hranice Ltd., Hranice, Czech Republic). The powder density was then obtained from the known mass of the sample in cylinder and its volume. Thermal parameters of aggregates were determined using the commercially produced device ISOMET (ISOMET 2114, Applied Precision Ltd., Bratislava, Slovakia) working on a transient impulse method principle. For the measurement of both natural and EPP aggregates, a needle probe impressed into the graduated cylinder with compacted aggregates was applied. The measurement accuracy was 5% of the reading +0.001 W/mK for the thermal conductivity in the range 0.015–0.70 W/mK, and 10% of the reading for the thermal conductivity ranging from 0.70 W/mK to 6.0 W/mK. The accuracy of the volumetric heat capacity was 15% of the reading +1 × 10^3^ J/m^3^K. In grain-size analysis, powder density and thermal parameter measurements, 3 samples were measured for both applied aggregates. Moreover, measurement of thermal parameters was repeated three times for a particular studied aggregate. 

Automatic helium pycnometer (Pycnomatic ATC, Thermo Scientific, Milan, Italy) with fully integrated temperature control with a precision of ±0.01 °C, and a real multi-volume density analyzer was used for EPP specific density measurements. The microstructure of EPP particles was also studied using a digital optical microscope (Dino-Lite AM7012MZT, Dino-Lite Europe/IDCP B.V., Naarden, The Netherlands) with a resolution of 5 Mpx.

#### 2.2.2. MOC Composite Testing

In testing hardened lightweight composite samples, a minimum of 5 specimens was examined. The number of samples was chosen for expected inhomogeneity of materials tested.

The workability of the fresh MOC composites mixes was evaluated by the flow table method in accordance with EN 12350-5 [30]. The flow measurement was done on 3 samples of fresh mixes.

Hardened MOC-R and MOC-EPP samples were characterized in terms of their basic physical and mechanical properties, thermal performance and water resistance. XRD, SEM, and EDS analyses were performed as well.

For the information of phase composition of MOC-based samples, XRD patterns were collected at room temperature on powder diffractometer (X’Pert^3^ Powder, PanAnalytical, Almelo, The Netherlands) with parafocusing Bragg-Brentano geometry using CuK_α_ radiation (*λ* = 1.5418 Å, U = 40 kV, I = 30 mA). The X’Pert HighScore Plus program was used for XRD data processing.

The microstructure and morphology of MOC-based composites were analyzed using scanning electron microscopy (SEM) (Lyra, Tescan, Brno, Czech Republic) with an FEG electron source (Tescan Lyra dual beam microscope). Energy dispersive spectroscopy (EDS) was performed with 20 mm^2^ SDD detector (X-Max^N^, Oxford instruments, Abingdon-on-Thames, UK) and AZtecEnergy software (Oxford instruments, Abingdon-on-Thames, UK). The accelerating voltage was 10 keV, and samples were placed on conductive carbon tape. The working distance for the measurement was between 5–7 mm. The pressure inside the vacuum chamber was lower than 0.1 Pa. Before the measurement, bulk samples were cut into 2 cm × 2 cm × 5 mm pieces and polished with fine grinding paper. Finally, gold sputtering was used (10 nm layer) to avoid charging. 

Before measurement of matrix and bulk densities, samples were first dried at 60 °C in a vacuum drier. Helium pycnometry (see above) was used for the matrix density determination. The bulk density was accessed on the gravimetric principle from dry sample mass and sample sizes following EN 12390-7 [31]. The expanded combined uncertainty of the bulk density test was 2.4%. The porosity was calculated on the basis of matrix and bulk density measurements. The relative expanded combined uncertainty of the porosity test was 3.5%.

Evaluation of mechanical resistance included compressive and flexural strength measurements and determination of Young´s modulus of elasticity for 28-day air-cured samples. Both strength tests were carried out according to the standard EN 14016-2 [32]. Prismatic samples with a dimension of 40 × 40 × 160 mm were used for determination of the flexural strength. The compressive strength was obtained on the broken halves of samples from the flexural strength test, where the loading area was 40 × 40 mm. In order to evaluate the strength development of MOC composites, the compressive strength of MOC-R and MOC-EPP samples was additionally measured after 3, 7 and 14 days of air-curing. The relative expanded uncertainty of the compressive and flexural strength tests was 1.4%.

Measurement of Young´s modulus of elasticity was done on a dynamic principle using the pulse ultrasonic method [33,34]. Before the ultrasonic measurement, the samples were dried in a vacuum drier at 60 °C until constant mass was achieved, i.e., the difference in sample mass after three independent measurements performed after 24 h of drying was less than 0.1% of the sample mass. In sample drying, we followed standard EN ISO 12571 [35]. An ultrasonic flaw detector (DIO 562, Starmans Electronics, Prague, Czech Republic) working on a frequency of 50 KHz was used, and the measurement was carried out on 40 × 40 × 160 mm prismatic samples in a longitudinal direction. The expanded combined uncertainty of the test method was 5.6%. 

Thermal conductivity *λ* (W/mK) and volumetric heat capacity *C_v_* (J/m^3^K) were measured on cubic samples with a side dimension of 70 mm to study the effect of the waste-based EPP aggregate on the improvement of heat transport and storage properties of the developed MOC composite. A commercially produced device ISOMET 2114 (see above) with a circular surface probe was used for the measurement. Details of the measurement accuracy are given above with respect to the aggregate characterization. Thermal properties of MOC composites were measured after 28 days of air curing (at 21 °C and 45% of relative humidity), without drying. At the time of measurement, the moisture content by mass *u* (%) of the MOC-R samples was 3% and 9.6% for the MOC-EPP samples. The moisture content was accessed on a gravimetric principle, from the measurement sample mass of wet samples (air-cured samples) and the mass of samples dried in a vacuum drier. For the measurement, digital laboratory balances that allowed measurement with an accuracy ±0.01% of the sample mass were used. Calculation of the moisture content was done according to the standard EN ISO 12570 [36].

For characterization of water resistance of the investigated MOC-based materials, cubic specimens were air-cured for 28 days (denoted A28) and part of the samples were then cured for another 28 days in tap water (denoted A28W28). For both groups of samples, the cubic compressive strength *f_c_* was measured after the respective treatment period. The water resistance coefficient *α_w_*, which indicates the relative variation of the strength of the hardened MOC-based composites with time in water, was then calculated according to Equation (1) [21].

(1) $$\alpha w=\frac{fcA28W28}{fcA28}$$

## 3. Results and Discussion

**Figure 4:**
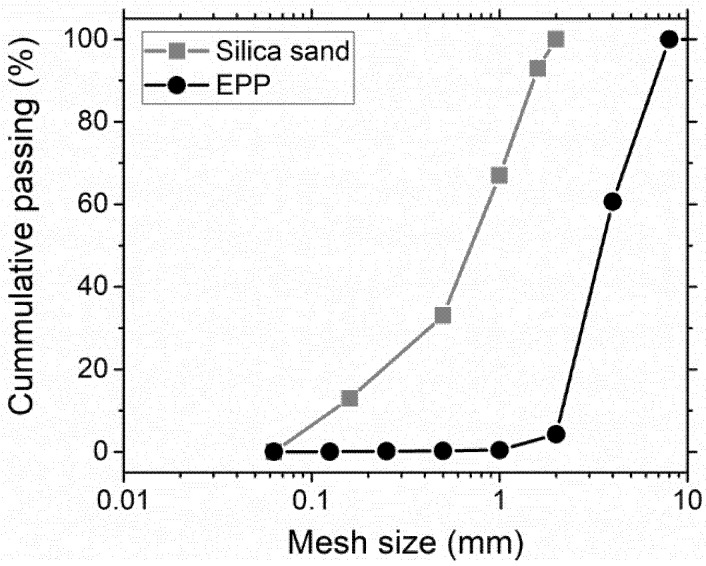
Grain-size curves of used mixed silica sand and EPP aggregates. The mesh size is presented on a logarithmic scale.

Figure 4 shows the grain-size curves of both natural and EPP aggregates. We can see that all EPP particles were smaller than 4 mm, which is suitable for their use as a silica sand replacement.

The specific density value of the EPP aggregate was 105 kg/m^3^. The EPP aggregate therefore belongs to artificial ultra-lightweight (density of less than 300 kg/m^3^) non-absorbent aggregates as given by Babu and Babu [37]. The thermophysical parameters of the EPP aggregate and silica sand as well as their corresponding powder density are summarized in Table 4. 

The EPP aggregate exhibited significantly lower heat transport than silica sand typically used in mortars and fine-grained concretes. The volumetric heat capacity of silica sand was observed to be markedly higher in comparison with EPP due to its high density and mineral origin. With increasing time of compaction, the thermal conductivity and volumetric heat capacity increased due to the elimination of air gaps between the particles. This aggregate behavior can be expected during preparation of MOC composites with incorporated EPP aggregate.

**Figure 5:**
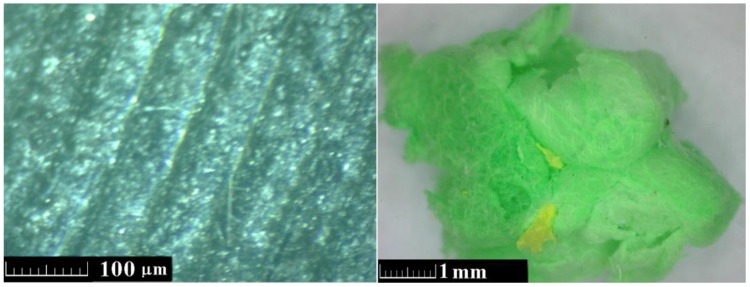
Optical microscopy images of the EPP particle.

Figure 5 displays images from the optical microscopy investigation under two different resolutions. The EPP foam consisted of closed-cell beads of high porosity that is linked to its low weight, high strength-to-weight ratio, low thermal conductivity and sound insulation properties. As the EPP aggregate used in this study comes from the crushing of waste EPP material, the obtained crushed particles has different colors, shapes and morphologies, but their skin texture is homogeneous and strongly oriented as apparent from the left microphotograph. 

As expected, the use of the EPP aggregate led to a reduction in workability from a flow diameter of 235 mm (MOC-R) to 140 mm (MOC-EPP). Although there is no literature on MOC composites containing recycled plastic aggregates, the fact that the incorporation of plastic aggregates leads to the decrease of workability is reported by many authors for composites with Portland cement used as a binder [13,38]. However, the resulting workability of the developed MOC-EPP composite is still suitable for the intended application of the developed material in floor structure, sandwich insulation panels, facade panels, etc. 

**Figure 6:**
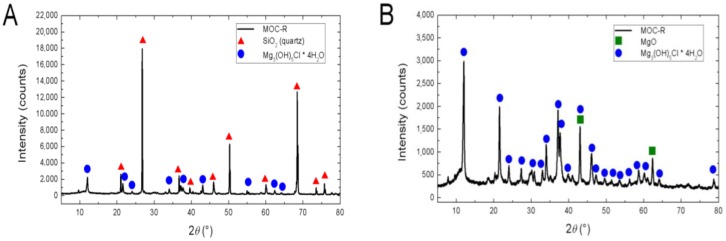
XRD diffraction pattern of (**A**) MOC-R and (**B**) MOC-EPP.

According to XRD (Figure 6A), phase composition of the sample MOC-R contained two phases: strongly diffracting quartz (JCPDS 01-085-0865) and Mg_3_(OH)_5_Cl·4H_2_O (JCPDS 00-007-0420). Also, a very low amount of MgO (JCPDS 01-077-2364) was detected (presence of MgO is not visible in the XRD diffractogram). Phase Mg_3_(OH)_5_Cl·4H_2_O can be also expressed as 5Mg(OH)_2_·MgCl_2_·8H_2_O. The second sample, MOC-EPP (Figure 6B), contained as a major phase Mg_3_(OH)_5_Cl·4H_2_O and also a minor phase of unreacted MgO (JCPDS 01-077-2364). The content of unreacted MgO was very low and can be assigned to the mix inhomogeneity and concentration of the MgCl_2_ solution that affected the mix workability. A similar small amount of residual MgO was observed, e.g., Yanni et al. [16].

**Figure 7:**
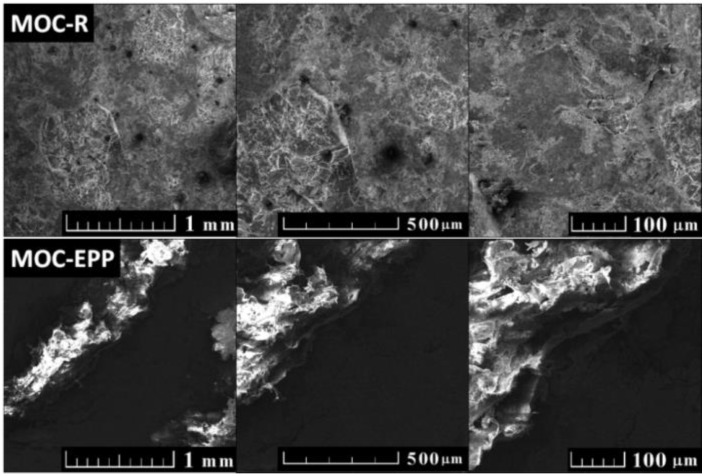
SEM micrographs of MOC-R and MOC-EPP at various magnifications.

Scanning electron microscopy (SEM) was used to analyze the morphology and binding conditions between the polymer and matrix (see Figure 7). Measurement was performed on cut surfaces after gold sputtering. In the case of MOC-R, a relatively flat surface without any cracks or major defects was examined. On the other hand, sample MOC-EPP was not entirely flat because plastic deformation occurred when cutting the polymer and now the polymer is above the surface of the matrix. Moreover, even gold sputtering in combination with low accelerating voltage of the electron beam was not sufficient to remove the charge from the polymer, thus the polymer is much brighter in comparison to the matrix. However, it can be seen that the polymer was retained very tightly in the matrix; even the cutting did not break the bond with the matrix.

**Figure 8:**
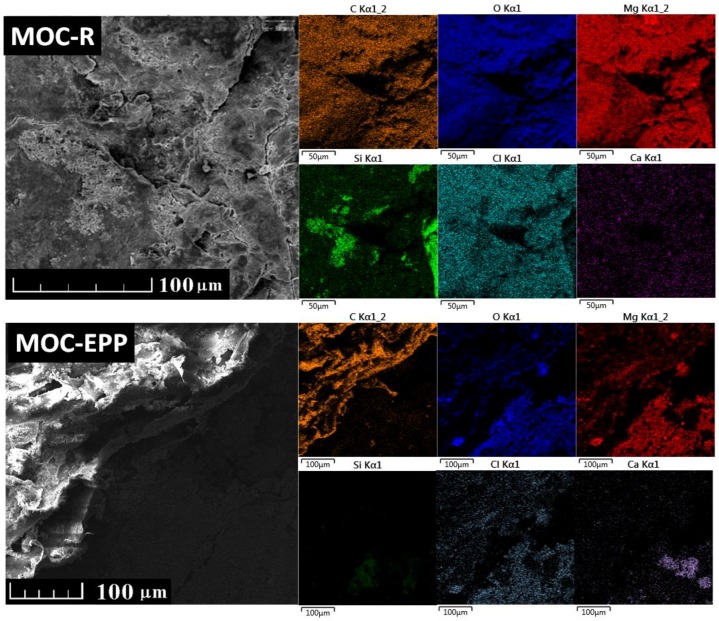
Elemental distribution maps of MOC-R and MOC-EPP obtained by EDS.

EDS maps are shown in Figure 8. For both samples, carbon, oxygen, magnesium, chlorine and calcium were detected. Silicon was also obtained for MOC-R; however, the silicon content in MOC-EPP was negligible. The obtained results confirmed the presence of 5Mg(OH)_2_·MgCl2·8H_2_O and quartz in MOC-R, which is in good agreement with XRD. In sample MOC-EPP, the chemical composition of the matrix is very similar in comparison to MOC-R; only SiO_2_ (quartz) was not present. According to the carbon elemental map, we can clearly recognize the polymer in the left upper corner in MOC-EPP.

Matrix density, bulk density and porosity data of the developed MOC composites are presented in Table 5. The presented data represent average values from 5 measured samples. 

We can see that incorporation of ultra-lightweight EPP aggregates into the MOC matrix led to a significant decrease in both matrix and bulk densities. The low bulk density of the MOC-EPP composite made it possible to classify it as lightweight concrete in class LC 1.0 according to EN 206-1 [39]. On the contrary, the presence of plastic particles caused a corresponding increase in porosity. These obtained physical property results are similar to those reported for Portland cement-based concrete containing plastic aggregates [40]. Gu and Ozbakkaloglu [12] in their comprehensive review stated that with increasing plastic substitution, the density of concretes decreased regardless of the type or size of used plastic aggregates because plastic aggregates are generally lighter than natural aggregates. 

Table 6 shows data on the mechanical resistance of the tested MOC composites. Within the flexural and Young’s modulus tests, 5 samples were measured. The compressive strength was measured on the 8 halves of broken prisms from the flexural strength test. Similar to Misra and Mathur [41], we observed for both MOC-R and MOC-EPP samples higher flexural strength and higher elastic modulus than would be expected when using Portland cement concrete of the same compressive strength. In our case, the flexural/compressive strength ratio is approx. 0.31 for MOC-R and 0.76 for MOC-EPP samples. For OPC concrete, the flexural/compressive strength ratio is usually 0.1–0.2, as reported, e.g., in [42,43]. The high flexural/compressive strength ratio of MOC-based composites can potentially obviate the need of fiber reinforcement and permits reduction in thickness slab, panels, etc. made of MOC. The mixture containing EPP particles exhibits significantly lower mechanical resistance than that with silica sand, nevertheless, this mechanical performance is quite sufficient for its intended use as a non-bearing material for building components. The worsening of mechanical properties of Portland cement-based concrete with varying amounts and types of plastic aggregates was observed widely [12,13,43,44,45,46,47]. It is quite apparent that the mechanical strength of concrete is highly influenced by the strength of the used aggregate. Brooks et al. [48] reported a decrease in the compressive strength for Portland cement composites containing expanded polystyrene (EPS), from 39.3 MPa for reference sample to 12.5 MPa for the sample containing 28 vol. % of EPS; the decrease in strength the authors assigned primarily to the introduction of the weak aggregate phase, which promotes stress cracks to initiate and propagate within the materials under mechanical loading. Similar material performance under a mechanical load can also be anticipated in our case. 

**Figure 9:**
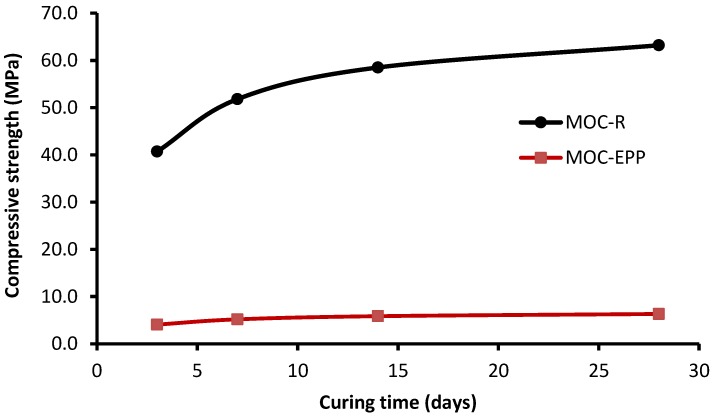
Compressive strength development of MOC-R samples.

The compressive strength development of MOC-R and MOC-EPP in time is shown in Figure 9. We can see rapid development of the compressive strength of both studied materials at the early curing age. From the 14^th^ day of curing, the compressive strength increased only slowly. This observation is consistent with the findings of Xu et al. [20].

The thermal properties of MOC-based materials air cured for 28 days are summarized in Table 7. The data present average values from 5 samples tested. 

Thermal conductivity can be regarded as one of the most important properties for construction materials considered for using in building envelopes for energy savings. The resulting thermal properties of MOC composites are mainly influenced by the composition and reactivity of the magnesium oxide powder, concentration of magnesium chloride solution, and the quantity and character of the aggregates used. As can be seen in Table 6, the use of EPP aggregates led to a great reduction in the thermal conductivity value that is almost 82% lower than for the reference sample. The contribution of the EPP aggregate with better thermal parameters compared to silica sand and increased porosity of MOC-EPP composite can be attributed as the main reasons for the observed thermal resistance improvement. The observed thermal conductivity of the MOC-R composite is consistent with the results of Xu et al. [20]. Brooks et al. [48] reported a decrease in thermal conductivity for Portland cement-based composites with incorporated EPS from 2.45 W/mK for the reference sample to 0.86 W/mK for the sample with 28 vol. % of EPS. Wang et al. [49] stated that the thermal conductivity of mortar with Portland cement as a binder containing high impact polystyrene (HIPS) was reduced to 87%, 69%, and 44% of the reference mortar when the HIPS ratio was 10%, 20%, and 50% by volume, respectively.

The water resistance of MOC composites was evaluated on the basis of the strength measurement. The water resistance coefficient was calculated from the cubic compressive strength of 28-day air-cured samples and from the retained compressive strength of the samples with 28 days prolonged exposure to water. The calculation was performed according to Equation (1). Within the water resistance tests, 5 samples of both studied MOC-based composites were analyzed. The obtained *α_w_* value was 0.68 for MOC-R and 0.28 for MOC-EPP sample. The lower *α_w_* value for the MOC-EPP material compared to the value measured for MOC-R can be assigned to the higher porosity of MOC-EPP samples and thus their higher water absorption capacity. The accessed water resistance coefficients were obtained for samples having a moisture content by volume of 5.9% (MOC-R) and 8.5% (MOC-EPP). Xu et al. [20] studied the influence of cenospheres on the properties of MOC-based composites and observed for reference samples a water resistance coefficient value 0.62. For samples with the addition of 15 and 20 mass % of cenospheres, the *α_w_* value decreased to 0.55 and 0.41, respectively. They assigned this strength degradation to the decomposition of phase 5 to brucite as a result of the hydrolysis reaction [50,51], which is known to have limited binding ability. As a similar degradation mechanism was anticipated in our case, after the leaching experiment, samples were analyzed using XRD. It was confirmed that unreacted MgO reacted with water and Mg(OH)_2_ was formed (brucite, JCPDS 00-001-1169). In addition, the more porous structure of the MOC composite with EPP facilitated water penetration in the composite matrix, which apparently led to an easier formation of brucite, and thus to more significant strength degradation. 

## 4. Conclusions

In this study, the influence of waste-based expanded polypropylene aggregate on the structural, mechanical, thermal and water resistance properties of magnesium oxychloride cement composites was investigated and evaluated. On the basis of obtained experimental results, the following conclusions can be drawn: 

The MOC matrix can incorporate a large amount of waste-based EPP aggregate, equivalent to 150% by volume of silica sand used in the reference composite, to still be usable in construction industry. Materials on the basis of MOC cement do not require water curing.

(1) The use of the EPP aggregate led to a 40.4% decrease in workability in comparison with the reference MOC-based material. Nevertheless, MOC-EPP exhibits sufficient rheological properties with good workability and placing characteristics.

(2) By incorporation of EPP, both the bulk and matrix densities of the hardened MOC composite decreased and porosity increased.

(3) Although compressive and flexural strength as well as Young’s modulus have been reduced by application of EPP aggregates, the MOC-EPP composite still maintains adequate mechanical parameters for intended use for non-structural thermal insulating applications. Generally, MOC composites have higher flexural strength and Young’s modulus as compared to Portland cement-based composites of the same compressive strength, even for composites containing plastic aggregates. MOC composites exhibit high compressive strength already at an early curing age.

(4) In comparison with silica sand, the EPP aggregate is characterized by significantly lower thermal conductivity. Consequently, for MOC-EPP composites, there was a great decrease of about 82% in the thermal conductivity value in comparison with the reference sample. 

(5) The obtained water resistance coefficient values confirmed the commonly recognized poor water resistance of MOC-based materials. Although MOC is sensitive to water, it can be exploited as a binder by adding chemical additives to improve its water resistance and thus was used also in applications, where contact with water cannot be avoided. 

It can be concluded that the application of waste-expanded polypropylene in magnesium oxychloride cement composites or/and MOC-based lightweight concretes can be considered as a promising option for the utilization of a large amount of this type of plastic waste. The developed MOC-EPP composite with improved thermal insulation properties can serve as a part of floor, ceiling, roof, wall or envelope components to reduce the energy consumption of buildings. Moreover, the developed MOC-EPP composite represent an environmentally friendly alternative to construction materials on a Portland cement basis. It should be also noted that MOC composites belong to air-dried materials; thus, appropriate design of building elements containing these composites must take into consideration their poor water resistance; possibly, additives improving MOC resistance against water must be used. 

## Figures and Tables

**Table 1:** Chemical composition of used MgO powder.

Substance (wt %)	
MgO	83
CaO	5
Fe_2_O_3_	1
SiO_2_	2
LOI	5

**Table 2:** Physical properties of used MgO powder.

Index	
Matrix density (kg/m^3^)	3344
Powder density (kg/m^3^)	843
Blain specific surface (m^2^/kg)	690

**Table 3:** Mix proportions of MOC-based composites.

Mixture	Mass (g)				
	MgO Powder	MgCl_2_ Solution	Silica Sand	EPP	
MOC-R	450	500	1350	-
MOC-EPP	450	500	-	21.8

**Table 4:** Thermophysical properties of aggregates with dependence on the compaction time.

Aggregate Type	Compaction Time (s)	Powder Density (kg/m^3^)	*λ* (W/mK)	*Cv* (× 10^6^ J/m^3^K)
EPP	0	18.9	0.042	0.049
	10	22.4	0.043	0.055
	30	23.3	0.044	0.058
	60	23.9	0.044	0.061
	180	24.3	0.047	0.062
Silica sand	0	1657	0.410	1.569
	10	1910	0.562	1.681
	20	1916	0.575	1.683
	30	1922	0.576	1.684
	60	1927	0.577	1.689

**Table 5:** Basic structural properties of MOC-based materials.

Material	Matrix Density (kg/m^3^)	Bulk Density (kg/m^3^)	Porosity (%)
MOC-R	2455	2124	13.5
MOC-EPP	1421	905	36.4

**Table 6:** Mechanical properties of MOC composites at 28 days of air curing.

Material	Compressive Strength (MPa)	Flexural Strength (MPa)	Young’s Modulus (GPa)
MOC-R	63.2	19.3	37.0
MOC-EPP	6.3	4.8	4.0

**Table 7:** Thermal conductivity and volumetric heat capacity of MOC composites.

Material	*λ* (W/mK)	*C_v_* (× 10^6^ J/m^3^K)
MOC-R	2.052	1.689
MOC-EPP	0.377	1.605
